# Music Healthful

**Published:** 1859-06

**Authors:** 


### MUSIC HEALTHFUL.

Music, like painting and statuary, refines, and elevates, and sanctifies. Song is the language of gladness, and it is the utterance of devotion. But coming lower down, it is physically beneficial; it rouses the circulation, wakes up the bodily energies, and diffuses life and animation around. Does a lazy man ever sing? Does a milk-and-water character ever strike a stirring note? Never. Song is the outlet of mental and physical activity, and increases both by its exercise. No child has completed a religious education who has not been taught to sing the songs of Zion. No part of our religious worship is sweeter than this. In David's day it was a practice and a study.

So wrote we a year or two ago, and with a long lost paternity, the paragraph has been going the round of the press ever since, showing that its truthfulness strikes the people. The most refreshing notes come from the human voice, and yet, skill in vocal music is the want of our nation; but good men and philanthrophic are waking up the attention of the people to this interesting subject, and it is becoming a regular exercise in our Sabbath and public schools.

On the admirable principles that what is worth doing at all, is worth doing well, and that to do anything well, one thing must be done at a time, we are glad to know that an Academy of Music has been in successful operation for ten years at Salem, Connecticut, under the presidency of the Hon. Orramel Whittlesey. Names high in the nation testify that it has been well and successfully conducted, and such being the case, it ought to overflow with pupils; and colonies like it should be established by its graduates in every state in the Union, and patronized by every family. With civil and religious liberty, with inherent material wealth beyond any nation on the globe, and a moral prestige equal to that of the highest, ours ought to be literally a nation of song; and gladness, and gratitude, and music, and mirth should daily make every habitation vocal with the concord of sweet sounds. A time so blessed, cannot come a day too soon.

